# Substituted Organotin Complexes of 4-Methoxybenzoic Acid for Reduction of Poly(vinyl Chloride) Photodegradation

**DOI:** 10.3390/polym13223946

**Published:** 2021-11-15

**Authors:** Angham G. Hadi, Sadiq J. Baqir, Dina S. Ahmed, Gamal A. El-Hiti, Hassan Hashim, Ahmed Ahmed, Benson M. Kariuki, Emad Yousif

**Affiliations:** 1 Department of Chemistry, College of Science, University of Babylon, Babylon 51002, Iraq; sci.angam.ganem@uobabylon.edu.iq; 2 Medical Laboratories Techniques Department, Al-Mustaqbal University College, Babylon 51002, Iraq; Dr.Sadiq_jaafer@mustaqbal-college.edu.iq; 3 Department of Medical Instrumentation Engineering, Al-Mansour University College, Baghdad 64021, Iraq; dina.saadi@muc.edu.iq; 4 Department of Optometry, College of Applied Medical Sciences, King Saud University, Riyadh 11433, Saudi Arabia; 5 Department of Physics, College of Science, Al-Nahrain University, Baghdad 10052, Iraq; hassan.hashim@nahrainuniv.edu.iq; 6 Polymer Research Unit, College of Science, Al-Mustansiriyah University, Baghdad 10052, Iraq; dr_ahmedabd@uomustansiriyah.edu.iq; 7 School of Chemistry, Cardiff University, Main Building, Park Place, Cardiff CF10 3AT, UK; kariukib@cardiff.ac.uk; 8 Department of Chemistry, College of Science, Al-Nahrain University, Baghdad 64021, Iraq; emad.yousif@ced.nahrainuniv.edu.iq

**Keywords:** poly(vinyl chloride), synthesis, 4-methoxybenzoic acid-tin complexes, irradiation, photodegradation, peroxide quenchers, hydrogen chloride scavengers, roughness factor

## Abstract

Poly(vinyl chloride) suffers from degradation through oxidation and decomposition when exposed to radiation and high temperatures. Stabilizers are added to polymeric materials to inhibit their degradation and enable their use for a longer duration in harsh environments. The design of new additives to stabilize poly(vinyl chloride) is therefore desirable. The current study includes the synthesis of new tin complexes of 4-methoxybenzoic acid and investigates their potential as photostabilizers for poly(vinyl chloride). The reaction of 4-methoxybenzoic acid and substituted tin chlorides gave the corresponding substituted tin complexes in good yields. The structures of the complexes were confirmed using analytical and spectroscopic methods. Poly(vinyl chloride) was doped with a small quantity (0.5%) of the tin complexes and homogenous thin films were made. The effects of the additives on the stability of the polymeric material on irradiation with ultraviolet light were assessed using different methods. Weight loss, production of small polymeric fragments, and drops in molecular weight were lower in the presence of the additives. The surface of poly(vinyl chloride), after irradiation, showed less damage in the films containing additives. The additives, in particular those containing aromatic (phenyl groups) substitutes, inhibited the photodegradation of polymeric films significantly. Such additives act as efficient ultraviolet absorbers, peroxide quenchers, and hydrogen chloride scavengers.

## 1. Introduction

Poly(vinyl chloride), PVC, is an important and common thermoplastic with many industrial applications. It has a high molecular mass and can be reshaped at relatively high temperatures. PVC has excellent performance and low cost, making it, therefore, a versatile candidate to replace many essential materials such as wood, steel, and glass [1,2]. PVC is highly involved in the production of construction materials (e.g., frames for windows and doors, cables, pipes, wall coverings, and flooring), medical products (e.g., bags for plasma and blood, infusion kits, and tubing), electronics (e.g., computers, laptops, keyboards, cords, and cellular phones), automobile components (e.g., dashboard skins, fabrics, and sealants), packaging (food bags, cling films, and bottles), plastic cards, clothing, office equipment, and sports materials [3]. The physical properties of PVC are dramatically altered when heated due to the changes at the glass transition and melting point [4]. High temperature, humidity, oxygen, water, and ultraviolet (UV) light cause PVC weathering [5].

The –CH_2_–CH(Cl) linkages within PVC do not absorb sunlight and are transparent at wavelengths (λ) higher than 220 nm. For terrestrial UV light, λ is above 290 nm. However, PVC suffers from photodegradation due to exposure to UV light at high temperatures. PVC photodegradation occurs mainly as a result of the presence of unusual bonds and chromophores (e.g., impurities) within the polymer [6,7]. PVC photodegradation results in the formation of chlorine radicals and unsaturated chains (polyenes). Chain scission (C-C) and the cleavage of C–Cl bonds in PVC leads to the formation of α-chloroalkyl radicals which couple together, leading to chain crosslinking [8]. Such a process results in yellowing and other surface discoloration, roughness, and the formation of a chalking thin layer that contains loosely adherent material [9,10]. In addition, volatile products, weight loss, and small polymeric residues ensue [11]. The presence of oxygen is vital in the photodegradation process of PVC, since it leads to chain scission auto-acceleration [12]. Therefore, high photostability is requisite for plastics used in construction materials, pipes, flame retardants, and other outdoor applications. The rate of PVC photodegradation can be manipulated using additives [13,14].

Many types of PVC additives have been developed, and the most common are plasticizers (e.g., phthalates, phosphates, and polyesters) to soften PVC by reducing its glass transition temperature, lubricants (e.g., lead and calcium stearates) to inhibit its adhesion, and fillers (e.g., metal carbonates and sulfates) to reduce its production cost and to enhance its hardness. In addition, PVC stabilizers (e.g., fatty acids, metal salts, and organometallics) are used to minimize PVC photodegradation [15,16,17]. Progress in the application of new photostabilizers to reduce PVC photodegradation continues and, in recent years, polybenzimidazoles [18], polyphosphates [19], pigments [20], metal oxides [21,22,23], Schiff bases [24,25,26,27,28], and organotin complexes [29,30,31,32] have been used.

Organotin compounds are easy to synthesize, and they have been used as intermediates in organic synthesis and other important applications [33,34,35]. For example, they act as biologically active reagents, wood preservatives, biocides, and miticides. The current work investigates the synthesis of new substituted tin complexes of 4-methoxybezoic acid and their application as PVC photostabilizers. In addition, the effect of the type of each substituent (aliphatic or aromatic) on the effectiveness of tin complexes is explored. 4-Methoxybenzoic acid is commercially available, stable, does not alter the color of PVC, and contains an aromatic moiety and heteroatoms. Therefore, this acid is a good ligand candidate for the formation of complexes with tin to produce potential PVC stabilizers.

## 2. Materials and Methods

### 2.1. General

Substituted tin chlorides (95–97%), 4-methoxybenzoic acid (99%), and solvents were supplied by Merck (Gillingham, UK). The PVC (MV = ca. 165,000, K-value = 67, degree of polymerization = 800) was supplied by Petkim Petrokimya (Istanbul, Turkey). Fourier transform infrared spectroscopy (FTIR) spectra were recorded on a Shimadzu FTIR 4200 spectrophotometer (Shimadzu, Tokyo, Japan). The melting points were recorded on a Gallenkamp apparatus (Calgary, AB, Canada). The element contents were determined using a Vario EL III elemental analyzer (Elementar Americas, Ronkonkoma, NY, USA). The ^1^H (500 MHz) and ^119^Sn nuclear magnetic resonance (NMR, 149 MHz) spectra were recorded on a Bruker DRX500 MHz spectrometer (Bruker; Zürich, Switzerland). An Ostwald U-tube viscometer (Ambala, India) was used to measure the viscosity of PVC solution in tetrahydrofuran (THF). The surface morphology of the PVC was inspected with a Meiji Techno microscope (Tokyo, Japan), an Inspect S50 microscope (FEI Company, Kohoutovice, Czech Republic), and a Veeco instrument (Plainview, NY, USA) to provide the optical, scanning electron microscope (SEM), and atomic force microscopy (AFM) images, respectively. The PVC weather impact was tested using a QUV acceleration weather-meter analyzer (Q-Panel Company, Homestead, FL, USA).

### 2.2. Synthesis of Complexes ***1*** and ***2***

**Scheme 1:**
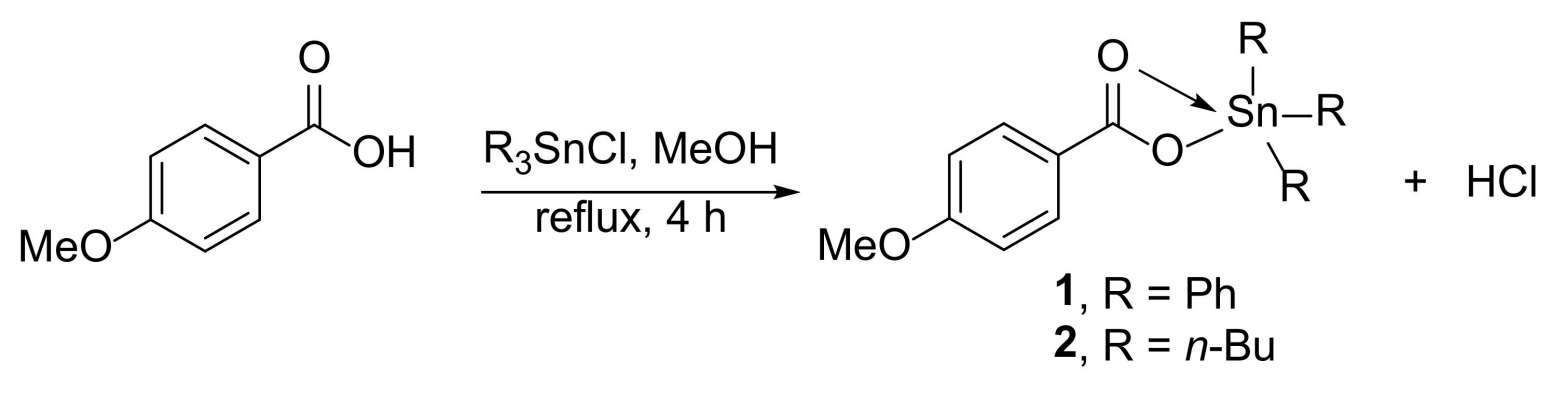
Synthesis of complexes **1** and **2**.

A solution of 4-methoxybenzoic acid (ligand; 0.76 g, 5 mmol) in methanol (MeOH; 50 mL) was added to a stirred solution of triphenyltin chloride (Ph_3_SnCl; 1.93 g, 5.0 mmol) or tributyltin chloride (Bu_3_SnCl; 1.63 g, 5 mmol) in MeOH (30 mL). The mixture was refluxed for 4 h and left to cool to room temperature. The solid obtained was filtered, washed with MeOH (2 × 20 mL), and dried to produce **1** or **2** (Scheme 1) in 78 or 71% yields, respectively (Table 1). The structures of **1** and **2** were confirmed by the FTIR and ^1^H NMR spectra and the elemental analysis for the carbon, hydrogen, and tin contents (Table 1).

### 2.3. Synthesis of Complexes ***3*** and ***4***

**Scheme 2:**
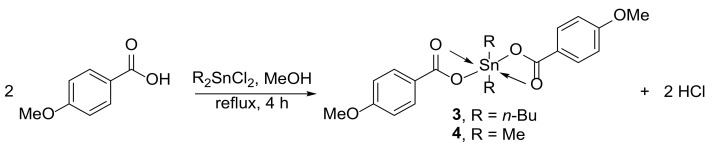
Synthesis of complexes **3** and **4**.

A solution of the ligand (0.91 g, 6 mmol) in MeOH (50 mL) was added to a stirred solution of dibutyltin dichloride (Bu_2_SnCl_2_; 0.91 g, 3.0 mmol) or dimethyltin dichloride (Me_2_SnCl_2_; 0.66 g, 3 mmol) in MeOH (30 mL). The mixture was refluxed for 4 h and left to cool to room temperature. The obtained solid was filtered, washed with MeOH (2 × 20 mL), and dried to produce **3** or **4** (Scheme 2) in 76 or 75% yields, respectively (Table 1). The structures of **3** and **4** were confirmed by the FTIR and ^1^H NMR spectra and the elemental analysis for the carbon, hydrogen, and tin contents.

### 2.4. Preparation of PVC Thin Films

Films of pure PVC and the material containing tin complexes of 4-methoxybenzoic acid were prepared based on the casting method at 25 °C [36]. The polymer (5 g) was dissolved in THF (100 mL) at 25 °C for 10 min. The Sn complexes (25 mg) were added to the PVC solution. The mixture was stirred for 2 h to ensure the complete homogeneity of the solution. The mixture was cast onto a glass dish (4 × 4 cm^2^) containing holes with a thickness of ca. 40 µm. The THF was evaporated at 25 °C for 16 h to produce a film at the bottom of the glass dish. The traces of solvent trapped within the blends were removed using a vacuum oven (16 h; 25 °C) to produce dry, thin films.

### 2.5. PVC Exposure to UV Light

The pure PVC and the samples blended with the Sn complexes of 4-methoxybenzoic acid were irradiated using a QUV tester. The UV light used had an intensity of 6.0 × 10^−9^ ein.dm^−3^.S^−1^ and a maximum wavelength (λ_max_) of 313 nm. The PVC films were rotated from time to time to ensure that the samples were exposed to UV light evenly from all sides. The time for the irradiation process was varied from 50 h to 300 h, in 50-hour intervals.

## 3. Results and Discussion

### 3.1. Synthesis of Complexes ***1***–***4***

The tin complexes of 4-methoxybenzoic acid **1**–**4** were synthesized as shown in Scheme 1 and Scheme 2 (Section 2.2 and Section 2.3). The reaction of an equimolar mixture of the ligand and Ph_3_SnCl or Bu_3_SnCl in refluxed MeOH gave the corresponding trisubstituted Sn complexed **1** or **2** in yields of 78 or 71% respectively (Table 1). Additionally, the reaction of Bu_2_SnCl_2_ or Me_2_SnCl_2_ (3 mmol) and the excess ligand (6 mmol) under identical conditions gave the corresponding Sn complexed **3** or **4** in yields of 76 and 75%, respectively (Table 1).

### 3.2. FTIR Spectroscopy of Complexes ***1***–***4***

The FTIR spectra of complexes **1**–**4** showed the absence of the bands corresponding to the stretching vibration of the O–H bonds that appeared in the 3100–2886 cm^−1^ region for the ligand as a result of the complexation with the Sn atom. The spectra contained two new sets of characteristic bands in the 459–472 cm^−1^ and 515–518 cm^−1^ regions due to the Sn–C and Sn–O bonds, respectively (Table 2). The asymmetric (*ν*asym) and symmetric (*ν*sym) vibrations of the carbonyl group in **1**–**4** appeared within the 1680–1683 cm^–1^ and 1508–1514 cm^–1^ regions, respectively (Table 2). The difference among the C=O asym and C=O sym vibration frequencies (Δ*ν*) were in the range 168–175 cm^–1^. Such small differences in the frequencies indicate chelating or bridging of the carboxylate group [37,38,39].

### 3.3. NMR Spectroscopy of Complexes ***1***–***4***

The ^1^H NMR spectra of Sn complexes **1**–**4** confirmed the absence of the carboxylic group proton (–CO_2_H) that appears as an exchangeable singlet at 12.23 ppm in the spectrum of the ligand. The observation was consistent with the complexation between the oxygen atom of the carboxylate group and the Sn atom. The ^1^H NMR spectra of **1**–**4** showed two doublets (*J* = 8.4–8.6 Hz) that appeared in the 7.97–7.64 ppm and 6.98–6.94 ppm regions (Table 3) due to protons of the aryl moiety. In addition, a singlet appeared at 3.84–3.78 ppm due to the presence of methoxy protons (OMe). The ^119^Sn NMR spectra of **1**–**4** showed singlet signals at −163 to −279 ppm, confirming that complexation had taken place between the ligand and the Sn atom. The chemical shift is dependent on the geometry of the complexes and the coordination number, which is believed to be either five or six [40,41].

### 3.4. Impact of Irradiation on Weight of PVC

Exposure of PVC to heat, light, and humidity leads to autocatalytic dehydrochlorination. The elimination of hydrogen chloride (HCl) causes discoloration, the formation of unsaturated fragments, drastic changes in mechanical and physical properties, weight loss, and a decrease in molecular weight. These undesirable changes are mainly due to cross-linking and chain scission [42,43]. Weight loss is indicative of the level of PVC photodegradation. Therefore, the PVC films were weighed before irradiation (*W*_0_), irradiated with UV light for different periods of time, and reweighed again at intervals of 50 h during irradiation (*W*_1_). Equation (1) was used to determine the weight loss (%) of pure PVC film and the films blended with the Sn complexes of 4-methoxybenzoic acid (40 µm) at different irradiation times.

(1) $$Weight\;loss\;\left(\%\right)=\left[\left(W_{0}\;–W_{1}\right)/W_{0}\right]\times 100$$

**Figure 1:**
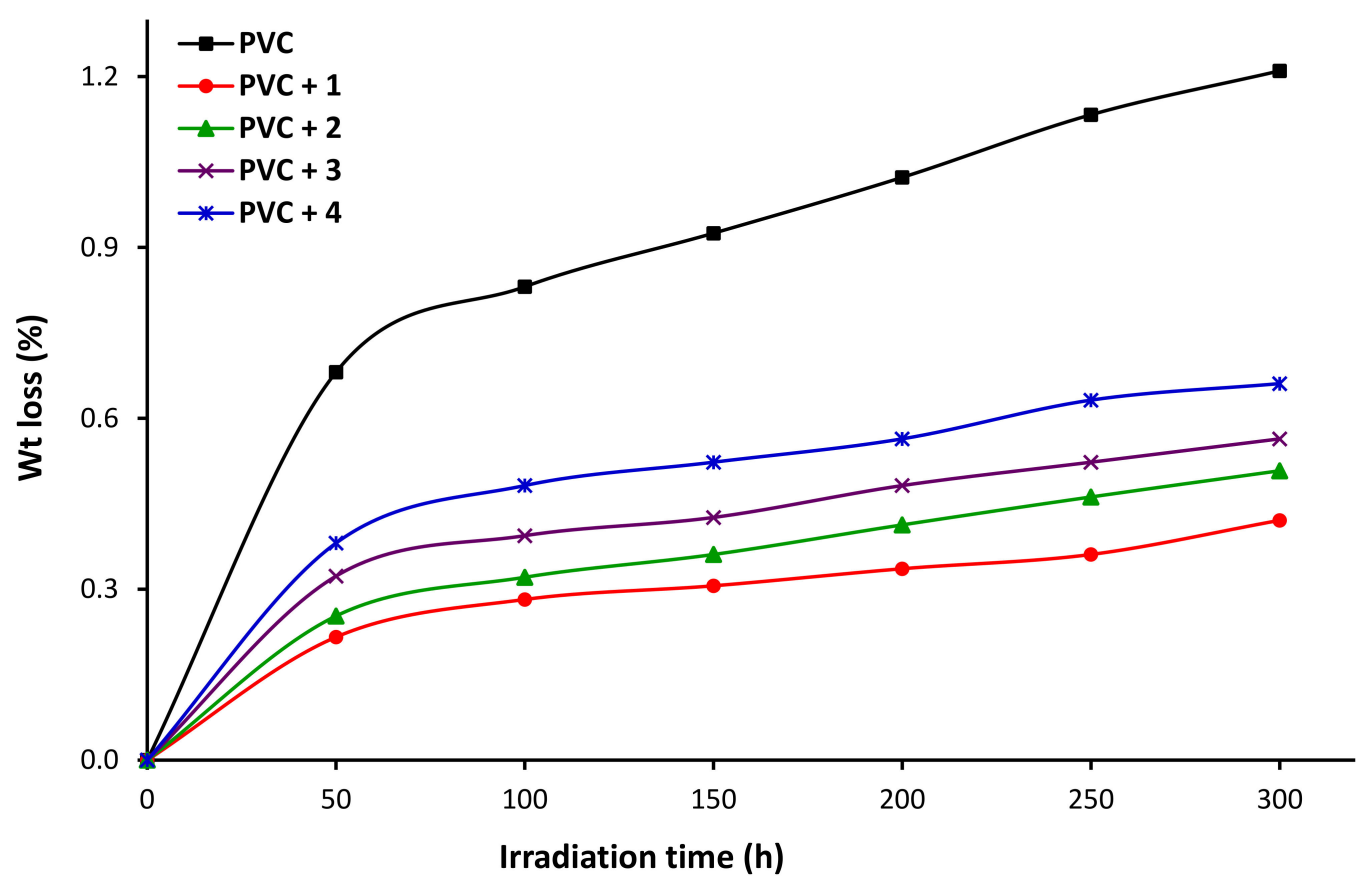
Percentage weight loss as a function of time during the irradiation of pure and tin-complex blended PVC.

The impact of irradiation time on the weight loss of the PVC films is shown in Figure 1. The additives undoubtedly played a significant role in stabilizing the PVC. The weight loss was fast during the first 60 h, and it increased steadily thereafter. The highest weight loss was observed in the case of the blank PVC film. The films blended with the Sn complexes led to lower weight loss compared with the pure film. The lowest weight loss was for the PVC film blended with triphenyltin complex **1**. Thus, the weight losses at the end of the irradiation process was 1.21% (pure PVC), 0.42% (PVC + **1**), 0.51% (PVC + **2**), 0.56% (PVC + **3**), and 0.66% (PVC + **4**). The greater stabilizing effect of complex **1** was ascribed to resonance and direct absorption of light associated with the aromatic rings [28]. The order of the efficiency of the PVC additives was **1** (triphenyltin) > **2** (tributyltin) > **3** (dibutyltin) > **4** (dimethyltin). The trend is in agreement with the order reported for other Sn complexes [29]. After 300 h, the films were physically damaged and turned dark brown, so no attempts were made to study the effect of irradiation beyond this period.

### 3.5. Impact of Irradiation on FTIR Spectroscopy of PVC

**Figure 2:**
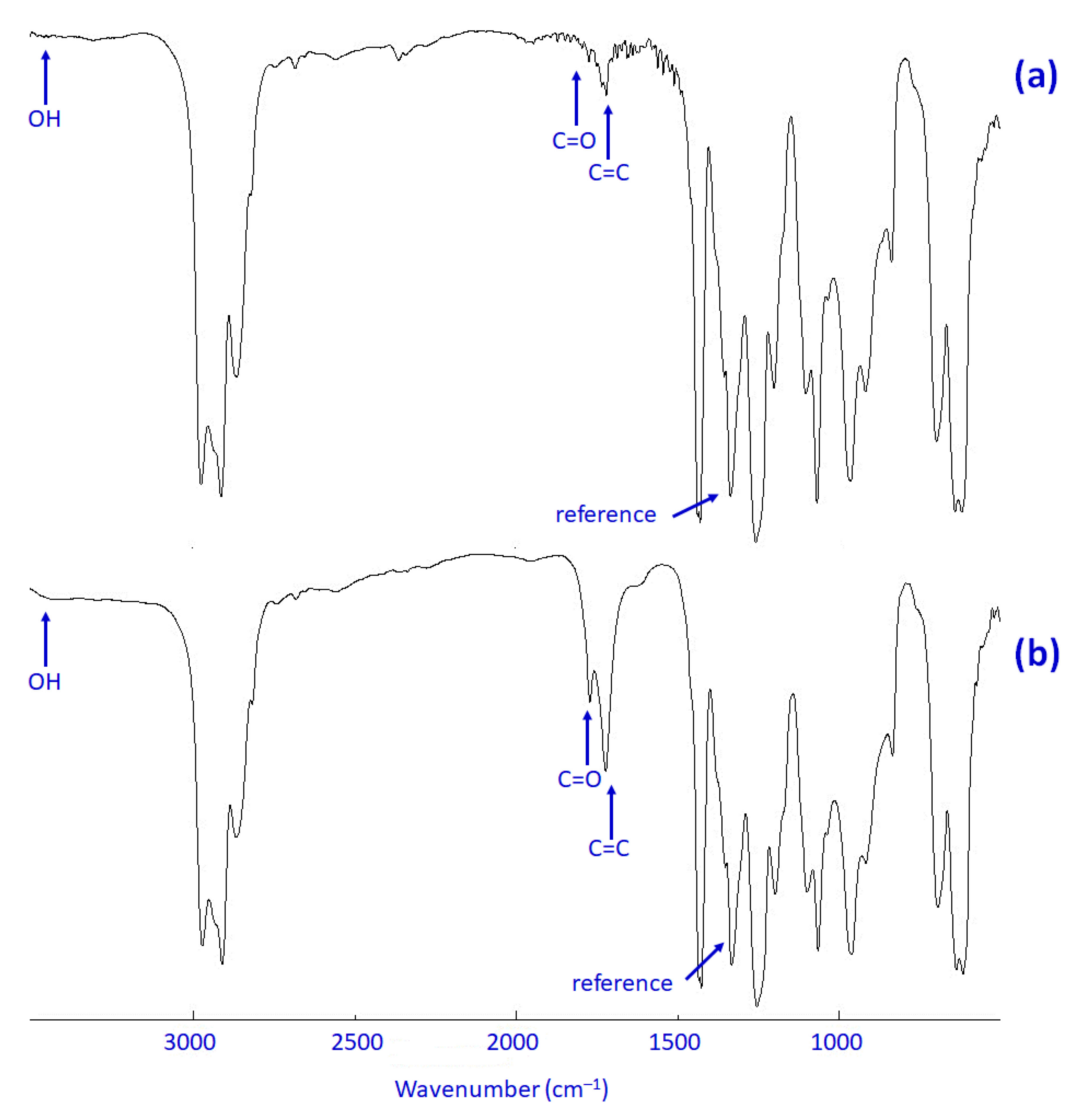
FTIR spectra of pure PVC (**a**) before and (**b**) after irradiation.

Elimination of HCl from PVC leads to the formation of unsaturated (–CH=CH–) small fragments and highly unstable (free radicals) polymeric chains. In the presence of oxygen, the PVC fragments containing radicals produced oxygenated residues containing carbonyl (–C=O) and hydroxy (–OH) groups [44,45,46]. Therefore, FTIR spectroscopy was used to study the impact of irradiation time on the formation of residues containing the –CH=CH– (1602 cm^−1^), –C=O (1722 cm^−1^), and –OH (3500 cm^−1^) groups. The intensities of the bands associated with these groups can be monitored during irradiation and compared with a reference peak. The irradiation has no impact on the absorption peak (1328 cm^−1^) corresponding to the C–H bonds in the PVC, and therefore it can be used as a reference [47]. In the experiment, the intensity of the absorption peaks corresponding to the functional groups increased as a result of irradiation (Figure 2). The functional group (–CH=CH–, –C=O, and –OH) index (*I*_s_) was calculated from the absorbance of both the functional (*A*_s_) and reference (*A*_r_) groups using Equation (2). The absorbances (*A*) of both the functional and references groups were calculated from the transmittance (*T*) using Equation (3).

(2) $$I_{s}=A_{s}/A_{r}$$

(3) A=2−logT%

**Figure 3:**
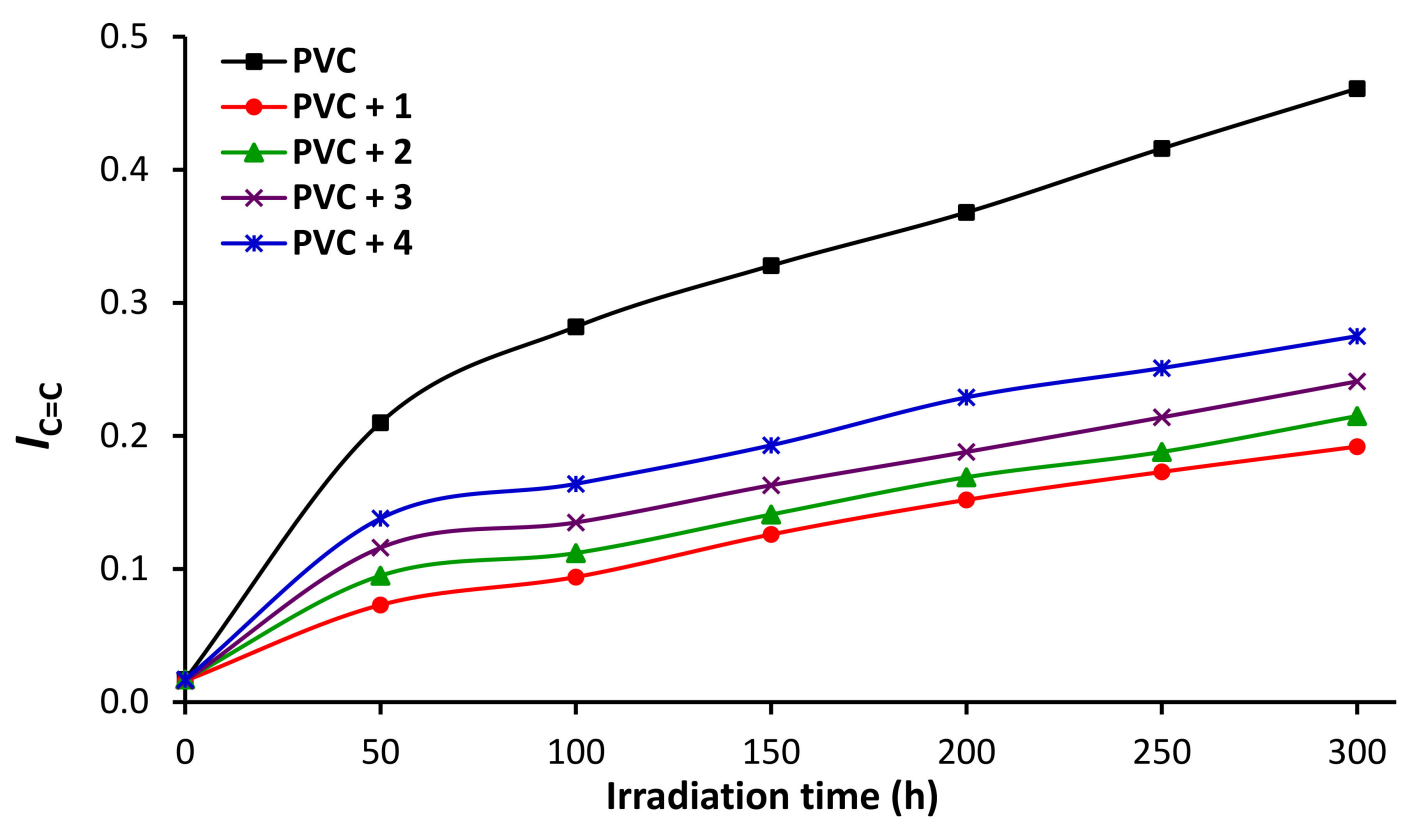
The *I*_C=C_ index as a function of time during the irradiation of pure and tin-complex blended PVC.

**Figure 4:**
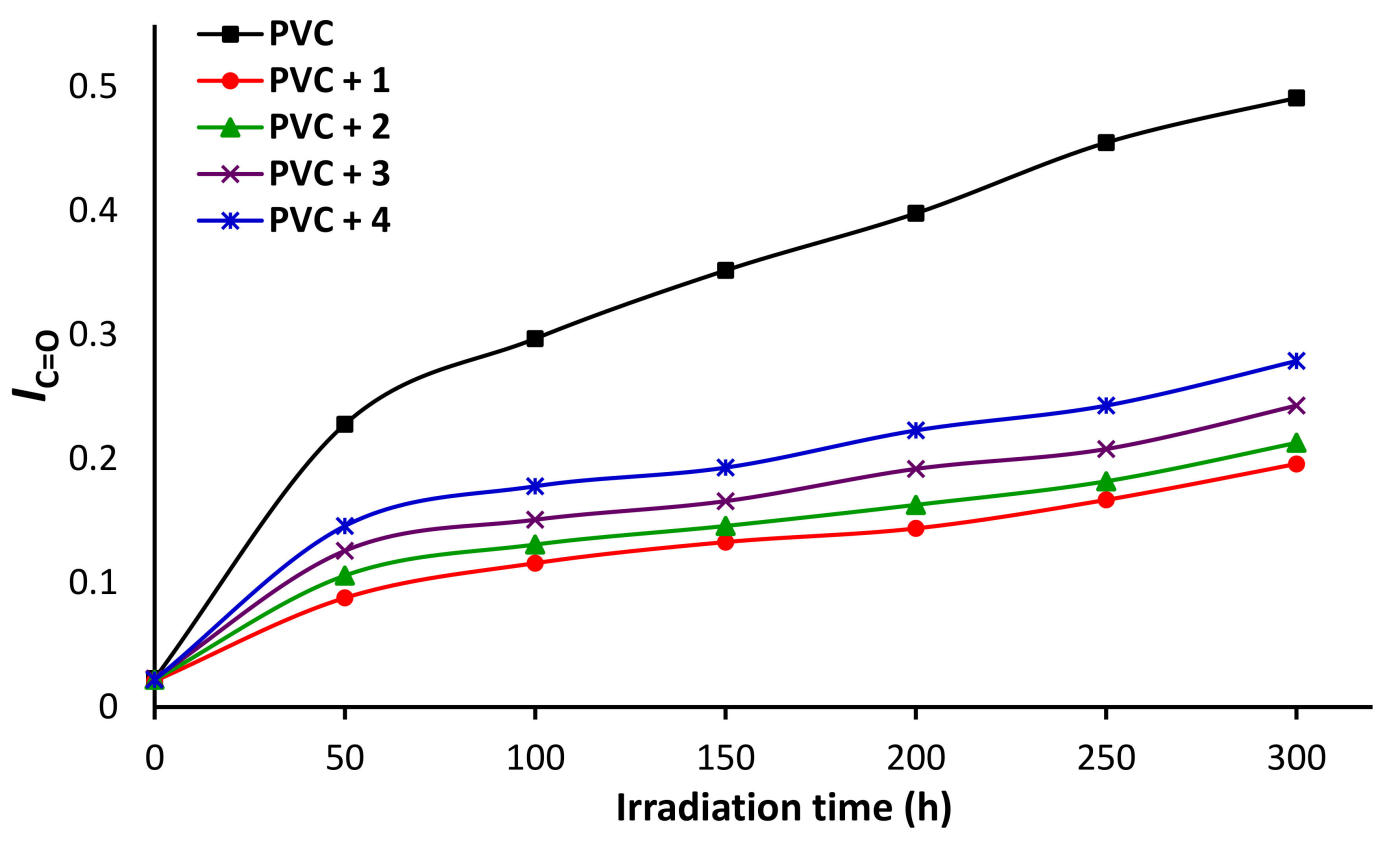
The *I*_C=O_ index as a function of time during the irradiation of pure and tin-complex blended PVC.

**Figure 5:**
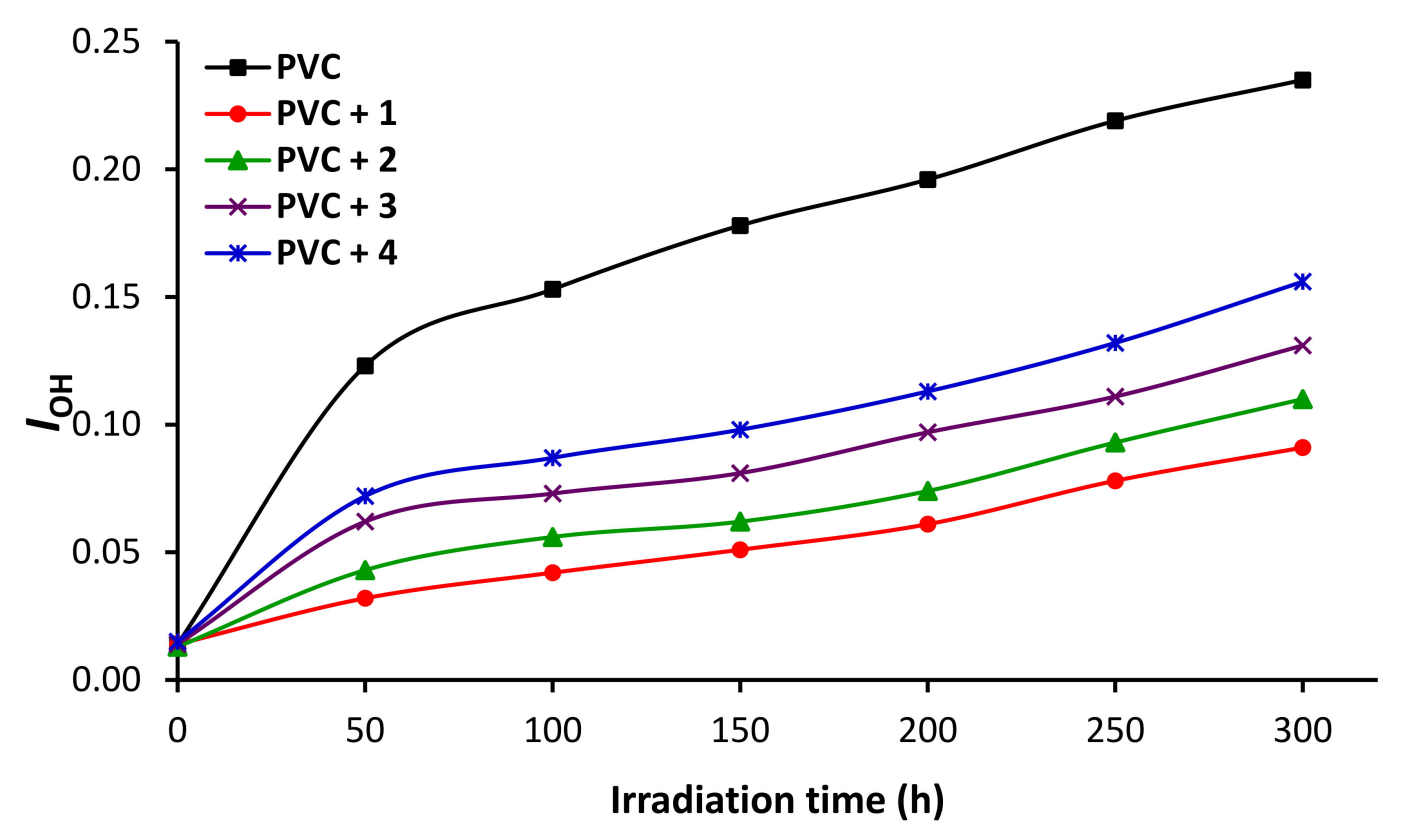
The *I*_OH_ index as a function of time during the irradiation of pure and tin-complex blended PVC.

The impact of irradiation on the indices of –CH=CH– (*I*_C=C_), –C=O (*I*_C=O_), and –OH (*I*_OH_) are shown in Figure 3, Figure 4 and Figure 5, respectively. The changes in *I*_C=C_, *I*_C=O_, and *I*_OH_ were less significant for the films containing Sn complexes. The results reflect the role played by the Sn complexes of 4-methoxybenzoic acid in reducing the impact of irradiation on the PVC films. The changes in the indices of the functional groups were fastest during the first 70 min and continued throughout the irradiation process. Complex **1**, with its high aromaticity, led to the least photodegradation. After 300 h of PVC exposure to UV light, *I*_C=C_ was 0.19 (PVC + **1**), 0.21 (PVC + **2**), 0.24 (PVC + **3**), 0.27 (PVC + **4**), and 0.46 (blank PVC). Similar results were also seen for *I*_C=O_ and *I*_OH_.

### 3.6. Impact of Irradiation on Molecular Weight of PVC

**Figure 6:**
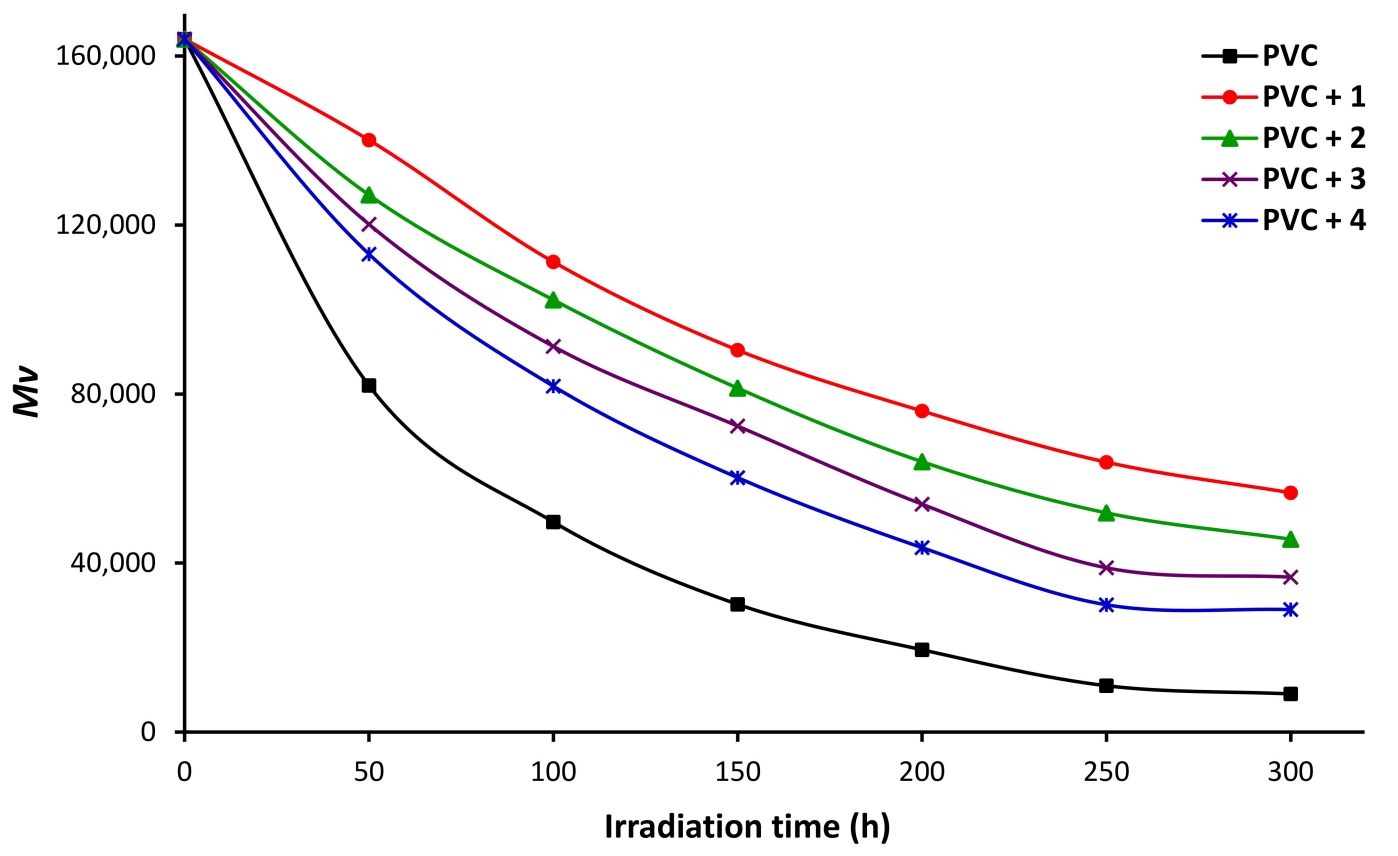
The molecular weight as a function of time during the irradiation of pure and tin-complex blended PVC.

PVC cross-linking and chain scission leads to the formation of small fragments. As a result, a decrease in the PVC molecular weight (${\bar{M}}_{V}$) is observed [48]. The decrease in the ${\bar{M}}_{V}$ is directly proportional to the PVC intrinsic viscosity [*η*]. A large [*η*] is indicative of a high degree of PVC damage due to photodegradation [49]. Following the irradiation of the PVC for different durations, the films were dissolved in THF and the viscosities of the solutions were measured using a viscometer. The changes in the ${\bar{M}}_{V}$ were calculated using the Mark–Houwink relation, as shown in Equation (4). The impact of irradiation time on the ${\bar{M}}_{V}$ is shown in Figure 6. In the absence of the Sn complexes of 4-methoxybenxoic acid, the decrease in the ${\bar{M}}_{V}$ was the sharpest of the samples. Thus, the blank PVC film lost 50.0% of its ${\bar{M}}_{V}$ after the first 50 h, 69.7% after 100 h, 88.1% after 200 h, and 94.5% after 300 h of irradiation. When Sn complexes **1**–**4** were used, the decrease in the ${\bar{M}}_{V}$ was much lower, with complex **1** being the most effective. For the PVC + **1** film, the loss to ${\bar{M}}_{V}$. was 14.6% after the first 50 h, 32.1% after 100 h, 53.7% after 200 h, and 65.5% after 300 h of irradiation.

(4) $$\left[\eta \right]=1.63\times 10^{-2}\;M_{v}^{0.77}$$

### 3.7. Impact of Irradiation on Surface Morphology of PVC

**Figure 7:**
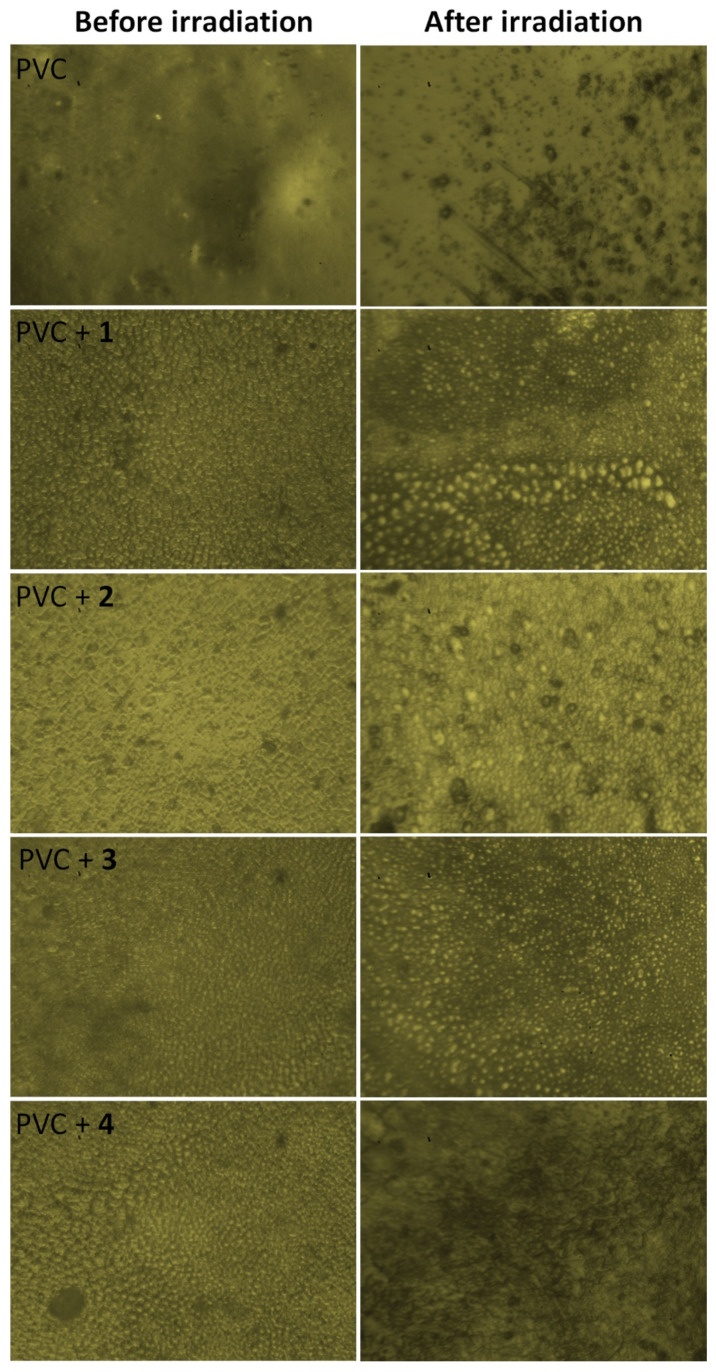
Microscopy images (400× magnification) of pure and tin complex-blended PVC.

Microscopy can provide useful information about crystallinity, irregularity, and defects within the surface of materials [50,51]. It can therefore be used to effectively monitor the surface roughness and appearance of cracks, dark spots, and other damage in PVC caused by photodegradation. Optical microscopy images of the PVC before irradiation showed very few dark spots and a smooth surface with no apparent cracks (Figure 7). The microscopy images for the irradiated pure PVC showed damage to the surface in the form of irregularities, dark spots, grooves, and cracks (Figure 7). The elimination of HCl is a major cause of PVC surface damage [52]. The damage was less apparent for the films containing Sn complexes of 4-methoxybenzoic acid, as the Sn atom aids the removal of HCl, reducing the surface damage. For the samples blended with the complex, the PVC surface irregularities were most noticeable in the case of the film containing complex **4**.

**Figure 8:**
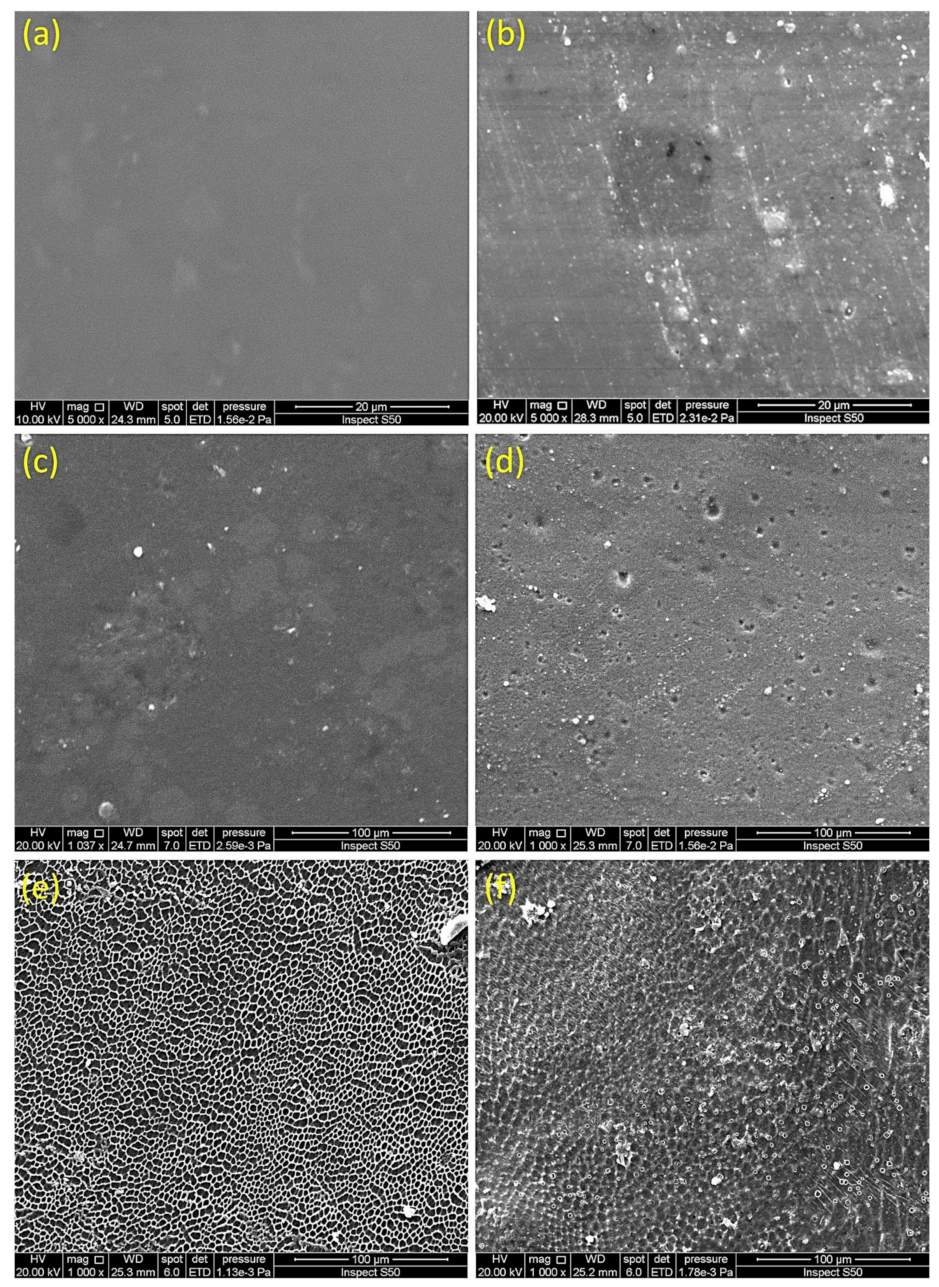
SEM images of (**a**) PVC before irradiation, (**b**) PVC after irradiation, (**c**) PVC + 1 after irradiation, (**d**) PVC + 2 after irradiation, (**e**) PVC + 3 after irradiation, and (**f**) PVC + 4 after irradiation.

**Figure 9:**
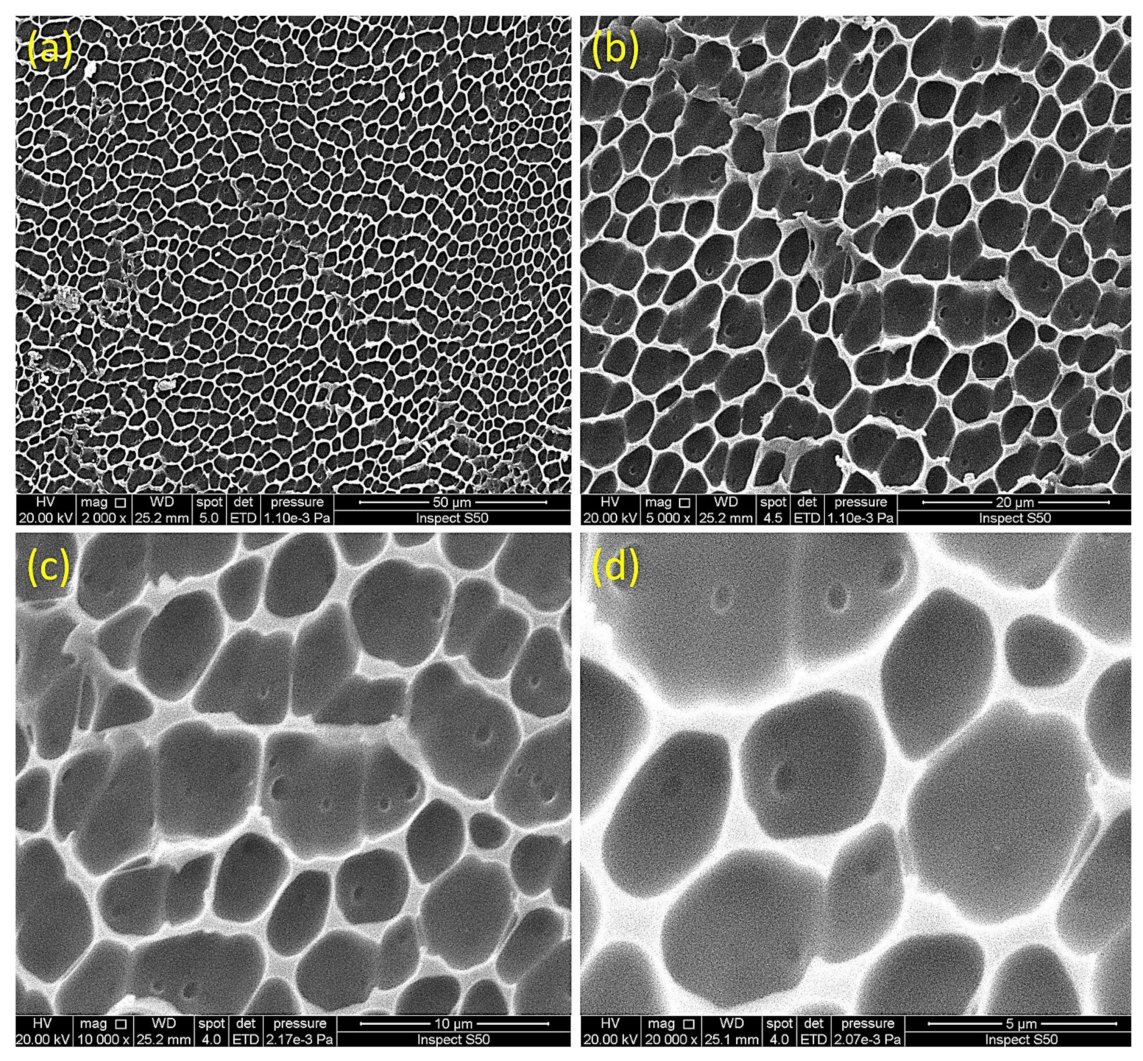
SEM images of PVC + **3** at magnification of (**a**) 2000×, (**b**) 5000×, (**c**) 10,000×, and (**d**) 20,000×.

The surfaces of the irradiated pure PVC and the blends were investigated further using SEM. SEM provides non-distorted, clear surface images and can record homogeneity, white spots, and the size and shape of particles [50]. The surface of the non-irradiated pure PVC film was homogeneous, smooth, and had no grooves or white spots (Figure 8a). In contrast, the SEM image for the irradiated pure PVC showed noticeable irregularity, white spots, groves, and lumps (Figure 8b), indicating a high level of photodegradation [53]. For the PVC blends containing Sn complexes of 4-methoxybenzoic acid, the surface irregularities and damage were greatly less than in the blank film (Figure 8c–f). This is consistent with the Sn complexes of 4-methoxybenzoic acid reducing the harmful effect of the released HCl on the surface of PVC. The SEM result agrees with the results from optical microscopy. The irradiated PVC films containing Sn complexes **3** and **4** showed a honeycomb-like structures. The honeycomb-like structure was clearly visible in the SEM images for the irradiated PVC + **3** film shown in Figure 9. The diameter of the pores within the PVC + **3** film ranged from 2.66 to 5.72 µm. A honeycomb-like structure has been reported previously after irradiation of PVC blended with a Schiff base containing a dithiazole moiety and nickel chloride [25,32]. In comparison, PVC film blended with a Schiff base containing a melamine unit led to the formation of an ice-rock-like structure [54]. In addition, the irradiation of PVC containing a trimethoprim Sn complex led to the formation of rod-like particles [55]. The reason for these phenomena is not clear, but they could be due to a slow rate of HCl elimination and its capture by the Sn complexes.

**Figure 10:**
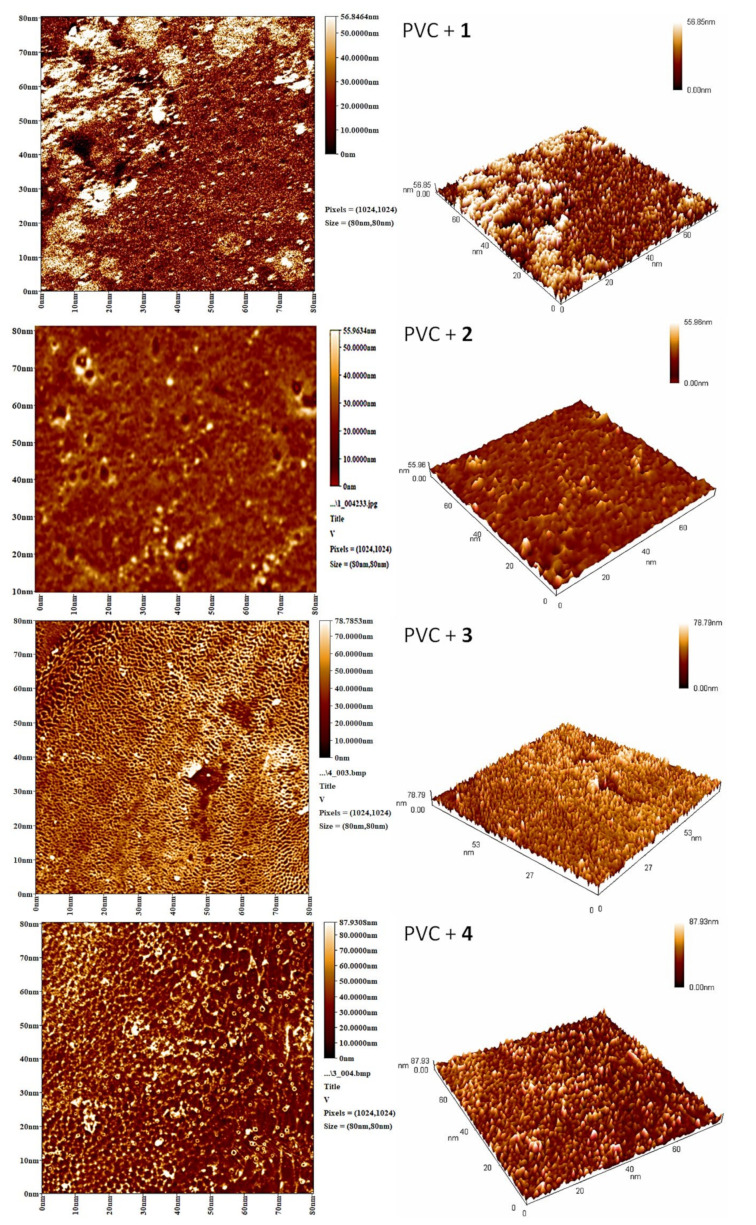
Atomic force microscopy (AFM) images showing the impact of irradiation on PVC–tin complex blends after irradiation.

The AFM technique was also used to investigate the smoothness of the irradiated PVC surfaces (Figure 10). The results showed that the occurrence of dark spots and roughness was more limited for the PVC films containing the Sn complexes. The roughness factor (*R_q_*) was 220.0 for the pure PVC, 10.4 for PVC + **1**, 13.6 for PVC + **2**, 14.2 for PVC + **3**, and 18.7 for PVC + **4**. Clearly, the Sn complexes reduced the *R*q significantly, which is further evidence for their role as PVC stabilizers. The Sn complex **1** reduced the *R*q by 21.2-fold compared with the blank film. Such a reduction in *R*q is remarkable and it was higher than those previously reported for other Sn complexes (Table 4) [56,57,58,59,60,61] and Schiff bases [62,63].

### 3.8. Impact of Sn Complexes on Photostabilization of PVC

**Figure 11:**
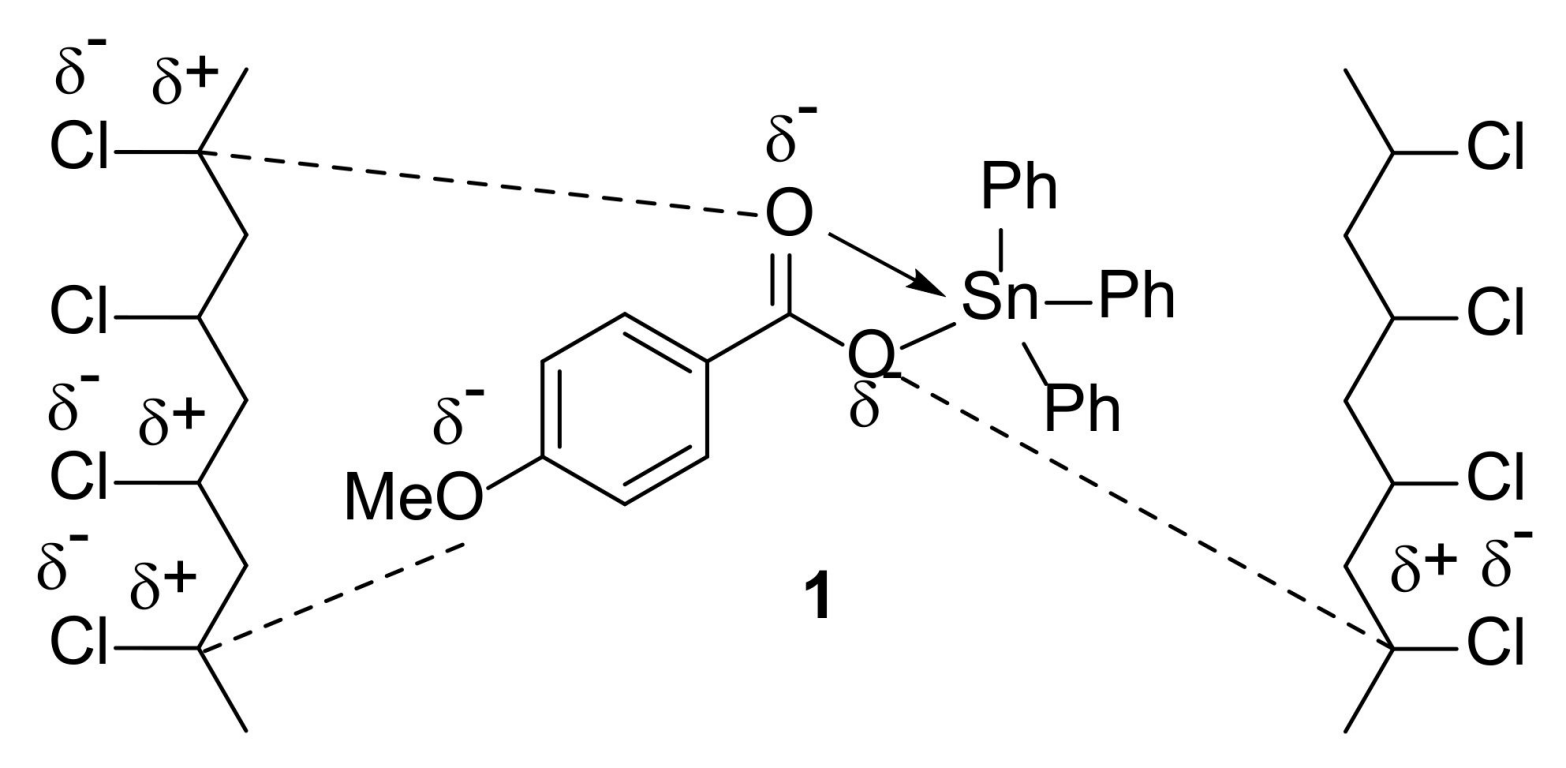
Coordination of **1** with PVC.

**Scheme 3:**
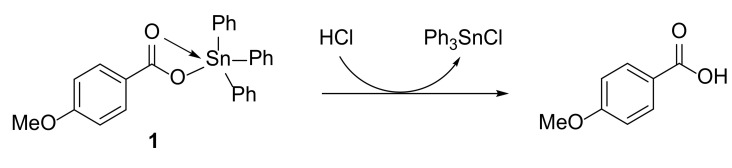
Complex **1** acts as a HCl scavenger.

**Scheme 4:**
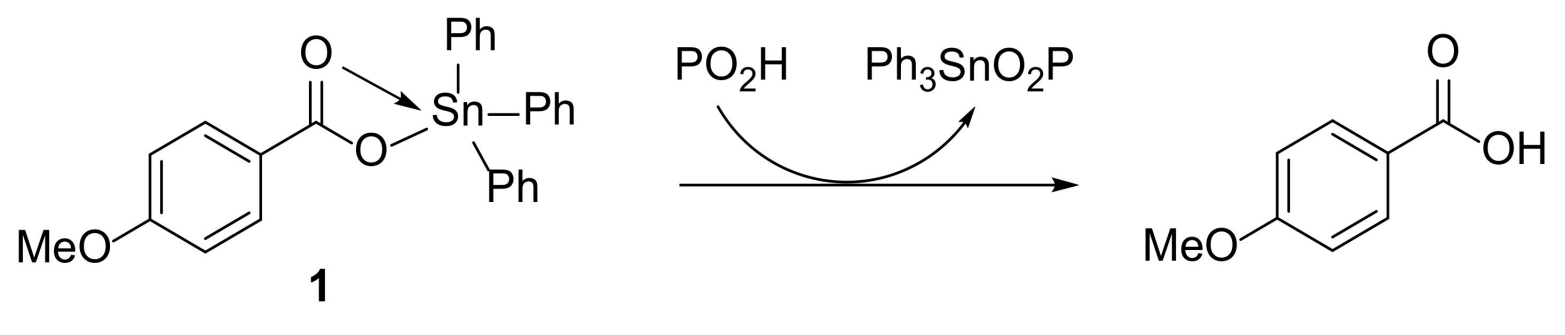
Complex **1** acts as a peroxide decomposer.

The Sn compounds **1**–**4** had a significant stabilizing effect, reducing PVC photodegradation. Various mechanisms for the role played by the Sn complexes have been suggested [57,58]. For example, the Sn complexes can coordinate with PVC chains (Figure 11) through polarized bonds and therefore stabilize the polymeric materials. The aromatic moieties in the Sn complexes, and in particular **1,** increased the stability of the excited state intermediate and led to a reduction in PVC damage [64]. The Sn atom also acts as a secondary PVC stabilizer. It acts as an HCl scavenger, and therefore reduces the harmful effects on the polymeric chains (Scheme 3). In addition, Sn complexes act as hydroperoxide decomposers (Scheme 4) through interaction with oxygenated species (e.g., hydroperoxide), releasing 4-methoxybenzoic acid [65]. Moreover, peroxide radicals can be quenched due to interaction with the Sn complexes. Such interactions would lead to the formation of charge transfer-stable intermediates through the resonance of the aromatic rings [60,66].

## 4. Conclusions

New tin complexes were synthesized from the reaction of 4-methoxybenzoic acid and substituted tin chlorides. The structures of the tin complexes were established and the materials were evaluated as stabilizers for poly(vinyl chloride). The complexes acted as effective ultraviolet absorbers, peroxide quenchers, and hydrogen chloride scavengers. They led to a reduction in the damage caused in the polymeric materials from prolonged irradiation with ultraviolet light. The reduction in weight loss, molecular mass decrease, and production of polymeric fragments was very noticeable when the tin complexes were blended with the polymer. In addition, the additives reduced the damage that took place on the surface of poly(vinyl chloride) on irradiation. The complex containing phenyl groups (aromatic moieties) was superior to those containing butyl and methyl groups (aliphatic moieties).

## Figures and Tables

**Table 1:** Physical properties and analysis of complexes **1**–**4**.

Sn Complex	R	Color	Yield (%)	M.P. (°C)	Calculated (Found; %)		
					C	H	Sn
**1**	Ph	Off white	78	153–155	62.31 (62.42)	4.42 (5.48)	23.69 (23.52)
**2**	Bu	White	71	144–146	54.45 (54.55)	7.77 (7.80)	26.91 (26.75)
**3**	Bu	White	76	147–149	53.86 (53.94)	6.03 (6.12)	22.18 (22.04)
**4**	Me	Off white	75	136–138	47.93 (47.98)	4.47 (5.56)	26.32 (26.24)

**Table 2:** Fourier transform infrared FTIR spectral data of complexes **1**–**4**.

Sn Complex	FTIR, Frequency (*ν*, cm^−1^)					
	C=O			C=C	Sn–O	Sn–C
	asym	sym	Δ*ν* (asym – sym)			
**1**	1680	1508	172	1591	518	468
**2**	1682	1508	174	1595	515	459
**3**	1682	1514	168	1592	515	468
**4**	1683	1508	175	1596	515	472

**Table 3:** Nuclear magnetic resonance NMR spectral data of complexes **1**–**4**.

Complex	^1^H NMR	^19^Sn NMR
**1**	7.94 (d, *J* = 8.6 Hz, 2H, Ar), 7.66–7.32 (m, 15H, 3 Ph), 6.97 (d, *J* = 8.6 Hz, 2H, Ar), 3.84 (s, 3H, OMe)	–178
**2**	7.97 (d, *J* = 8.5 Hz, 2H, Ar), 6.94 (d, *J* = 8.5 Hz, 2H, Ar), 3.78 (s, 3H, OMe), 1.71 (t, *J* = 7.6 Hz, 6H, 3 MeCH_2_CH_2_C*H_2_*), 1.50 (quintet, *J* = 7.6 Hz, 6H, 3 MeCH_2_C*H_2_*),1.35 (sextet, *J* = 7.6 Hz, 6H, 3 MeC*H_2_*), 0.93 (t, *J* = 7.6 Hz, 9H, 3 Me)	–163
**3**	7.66 (d, *J* = 8.4 Hz, 4H, 2 Ar), 6.98 (d, *J* = 8.4 Hz, 4H, 2 Ar), 3.80 (s, 6H, 2 OMe), 1.55 (t, *J* = 7.6 Hz, 4H, 2 MeCH_2_CH_2_C*H_2_*), 1.42 (quintet, *J* = 7.6 Hz, 4H, 2 MeCH_2_C*H_2_*),1.30 (sextet, *J* = 7.6 Hz, 4H, 2 MeC*H_2_*), 0.83 (t, *J* = 7.6 Hz, 6H, 2 Me)	–279
**4**	7.64 (d, *J* = 8.5 Hz, 4H, 2 Ar), 6.97 (d, *J* = 8.5 Hz, 4H, 2Ar), 3.79 (s, 6H, 2 OMe), 0.75 (s, 6H, 2 Me)	–269

**Table 4:** Reduction in the roughness factor *R*q (by fold) of PVC films in the presence of various Sn complexes.

Organic Moiety in Sn Complex	Reduction in *R*q (Fold)	Reference
4-Methoxybenzoic acid	21.2	[current work]
Naproxen	5.2	[56]
Carvedilol	6.4	[54]
Furosemide	6.6	[57]
Valsartan	7.4	[58]
Telmisartan	9.4	[59]
Trimethoprim	11.3	[55]
Ciprofloxacin	16.6	[60]
4-(Benzylideneamino)benzenesulfonamide	18.4	[61]

## Data Availability

Data are contained within the article.
